# EMS-Breathz: Development of an Electrical Muscle Stimulation (EMS)-Guided Rhythmic Breathing System to Actively Regulate Breathing Patterns During Radiotherapy

**DOI:** 10.7759/cureus.102989

**Published:** 2026-02-04

**Authors:** Kohei Oguma, Atsuya Takeda, Takafumi Nemoto

**Affiliations:** 1 Department of Radiology, Keio University School of Medicine, Shinjuku-ku, JPN; 2 Cancer Center, Keio University School of Medicine, Shinjuku-ku, JPN

**Keywords:** breathing pattern regulation, electrical muscle stimulation (ems), radiotherapy, respiratory motion management, rhythmic breathing

## Abstract

We developed EMS-Breathz, an electrical muscle stimulation (EMS)-guided rhythmic breathing system that regulates the breathing pattern during radiotherapy by inducing abdominal muscle contractions through rhythmic electrical stimulation. EMS-Breathz promotes exhalation during stimulation and passive inhalation during nonstimulation, thereby enabling repeatable respiratory cycles with minimal voluntary effort. The prototype functioned as designed in technical testing, demonstrating the feasibility of external respiratory modulation. By stabilizing breathing, this approach may reduce tumor motion uncertainty, improve respiratory gating accuracy, and help regulate patient breathing patterns. This technical report summarizes the system design, operation, and potential clinical applications.

## Introduction

Respiratory motion is one of the major factors that cause dose delivery uncertainty to the thoracic and abdominal tumors in radiotherapy [1]. During normal respiration, tumor movement can reach 20-30 mm, occasionally exceeding 50 mm, which can lead to dose deviations of 10%-30% when not properly managed [1,2]. When breathing becomes irregular, treatment sessions can become less accurate, causing a reduction in tumor coverage and unintended irradiation to normal tissues. In addition, extra time is required to return to a steady breathing rhythm or to rebuild the breathing prediction algorithm. To address these issues, various respiratory motion management systems have been developed, including external surrogate-based motion monitoring and assistance [3-6], breath-hold [7,8], and tumor-tracking systems [9,10]. However, these approaches primarily respond to or compensate for respiratory motion, rather than actively regulate the breathing pattern itself.

To overcome these drawbacks, we focused on electrical muscle stimulation (EMS) as a semiforced and external approach for regulating the breathing pattern. Unlike conventional guided breathing techniques that rely primarily on patient cooperation and voluntary respiratory control, EMS provides direct physiological modulation of respiration by externally inducing expiratory muscle contraction. EMS is a noninvasive method that induces muscle contractions by delivering controlled electrical pulses through surface electrodes [11,12]. The abdominal muscles, including the rectus abdominis, play an important role as expiratory muscles [13]. When applied to the abdominal muscles, EMS induces contractions during stimulation, naturally promoting exhalation and relaxation during the nonstimulation phase, leading to passive inhalation. Clinical studies have also demonstrated that abdominal EMS can enhance expiratory function and improve breathing patterns in patients with impaired respiratory control, including those with spinal cord injury [14] and chronic obstructive pulmonary disease [15].

To implement these mechanisms, we developed EMS-Breathz, an EMS-guided rhythmic-breathing system. By externally stabilizing each patient’s respiratory rhythm, the system aims to ensure more stable, reproducible dose delivery to the tumor, representing a novel strategy to reduce dose uncertainty caused by respiratory motion in radiotherapy while shortening treatment time.

## Technical report

System overview

**Figure 1:**
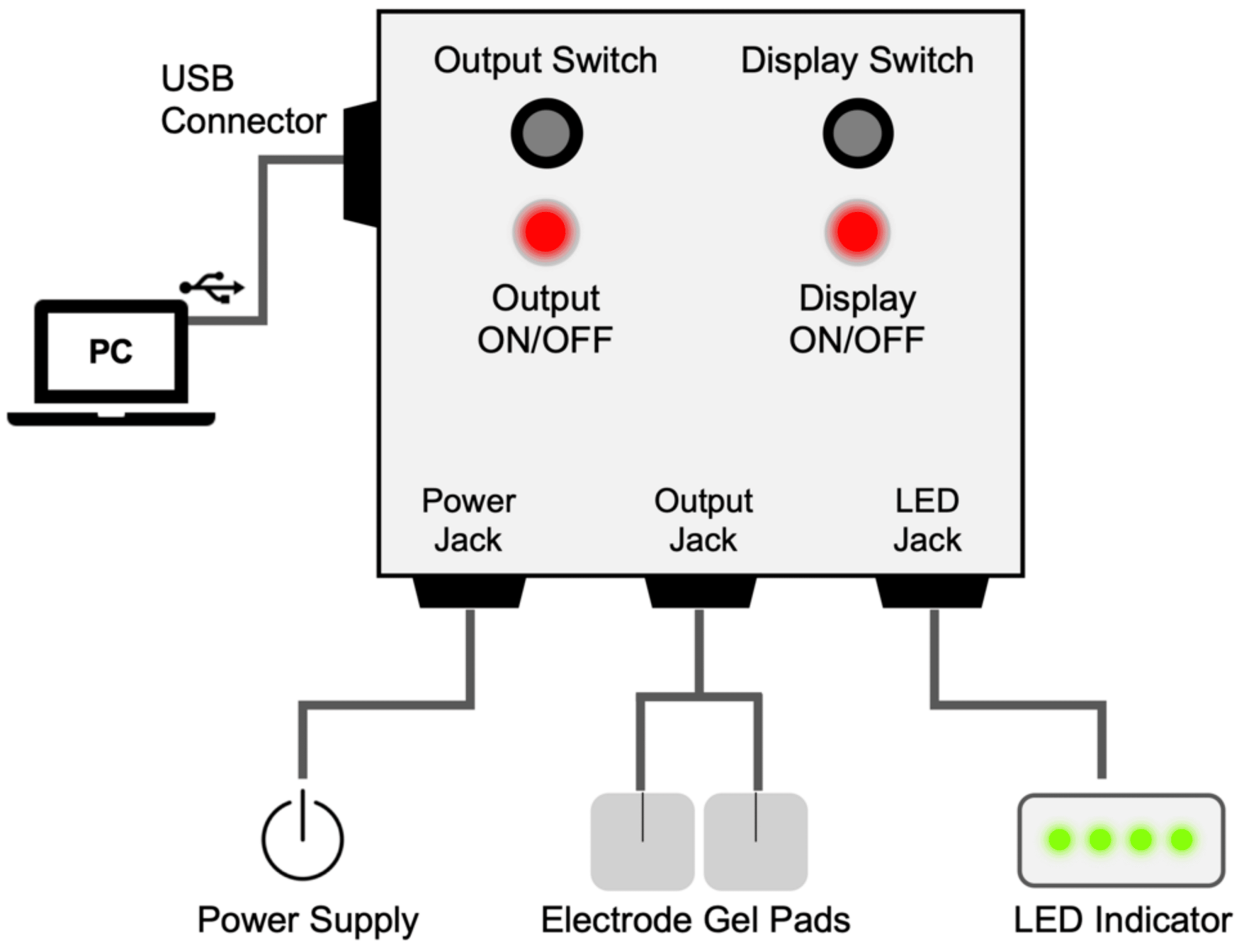
Hardware configuration of the EMS-Breathz stimulator unit LED: light-emitting diode; EMS: electrical muscle stimulation

**Figure 2:**
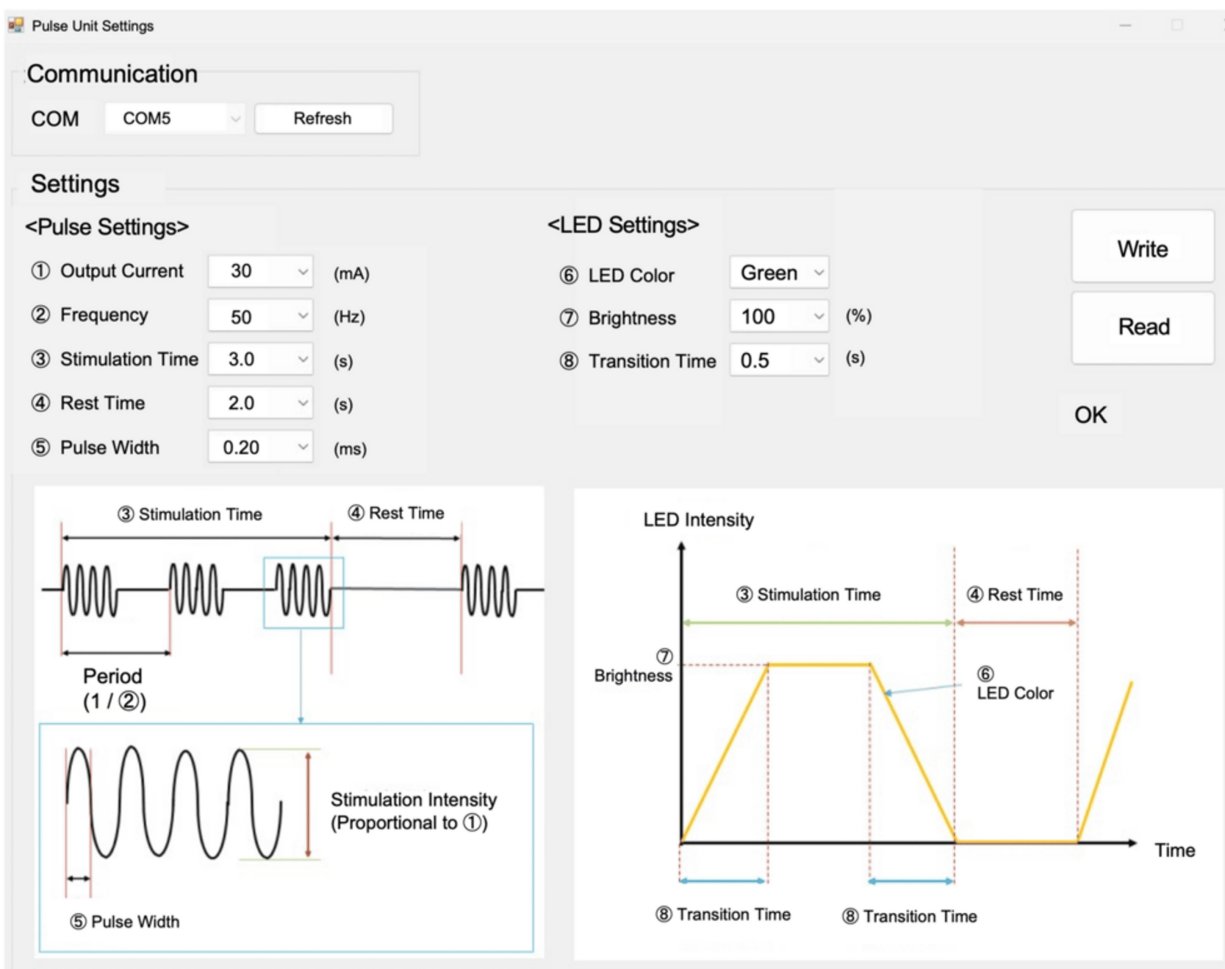
Graphical user interface for controlling stimulation and LED parameters LED: light-emitting diode

The EMS-Breathz consists of three main components: a stimulator unit, surface electrodes, and a graphical user interface (GUI) application. A schematic of the system configuration is shown in Figures 1, 2.

Stimulator unit

The stimulator generates biphasic electrical pulses with user-defined parameters (Table 1) and allows fine adjustment of output characteristics within the range typically employed for commercially available surface-electrode stimulation devices. Control signals are produced by an internal microcontroller and transmitted through electrically isolated circuits to ensure patient safety and prevent current leakage. The electronic components were designed with electromagnetic shielding to minimize potential interference in the radiotherapy treatment environment. The stimulator connects to a laptop computer via a USB interface and features light-emitting diode (LED) indicators that display output status in real time.

**Table 1:** User-defined stimulation parameters and adjustable ranges N/A: not available

Parameter	Unit	Range	Increment
Output current	mA	20-80	1
Output voltage	V	Up to 40	N/A
Frequency	Hz	30-120	1
Pulse width	ms	0.2-0.3	0.01
Stimulation time	seconds	0.1-10	0.1
Rest time	seconds	0-10	0.1

Surface electrodes

Two commercially available self-adhesive electrodes, Ebullient Compatible Gel Sheets EMS Replacement Pads (Ebullient, Japan), were placed on the abdominal wall along the orientation of the abdominal muscle fibers. Electrical stimulation induced mild to strong contractions of the rectus abdominis and oblique muscles according to the strength of the electrical output current. Electrode placement was flexible and could be adjusted based on patient anatomy or immobilization setup, with the skin cleaned beforehand with an alcohol swab. If hair was present at the application site, the electrode was placed on an alternative area, or the hair was shaved to ensure adequate electrical contact. Lead cables were routed to prevent interference with imaging or treatment beams. Single-use conductive gel pads were individually labeled and replaced for each patient to maintain hygiene.

Operation principle

The GUI application allows the operator to set stimulation parameters such as pulse width, frequency, output current, stimulation time, and rest time (Figure 2). During the stimulation phase, EMS output triggers abdominal muscle contractions, thereby promoting exhalation. During the nonstimulation phase, stimulation ceases, allowing passive inhalation. This cycle is repeated, generating an EMS-guided breathing pattern that helps patients maintain regular breathing without conscious effort. While consulting with the patient, the operator adjusts stimulation intensity and timing to match each patient’s baseline respiratory rhythm, which is measured and confirmed before treatment.

Results

**Figure 3:**
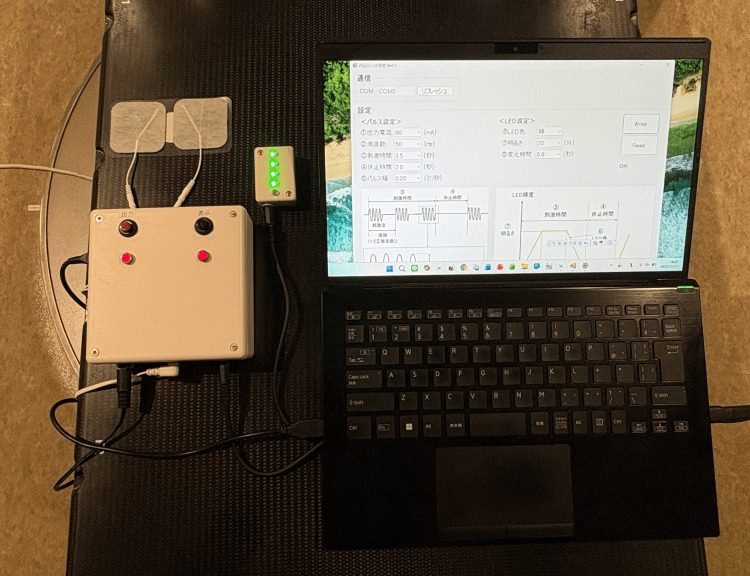
Prototype of the EMS-Breathz placed on a radiotherapy couch EMS: electrical muscle stimulation

The EMS-Breathz prototype was successfully developed in accordance with the intended design concept. Figure 3 shows the completed stimulator unit on a radiotherapy treatment couch. The compact control box (approximately 125 × 125 × 45 mm^3^) integrates the stimulator, control circuits, and interface components. Its front panel includes an output switch, status LEDs, output connectors, an external LED port, a power connector for an AC adapter, and a USB interface for PC connection. An external RGB LED module can also be connected to provide a visual indication of stimulation timing. An overcurrent protection circuit automatically interrupts output in the event of a short circuit, ensuring operational safety. The complete setup, comprising the GUI application and bilateral abdominal electrodes, delivered rhythmic electrical stimulation with adjustable timing and intensity. Stimulation patterns were easily customized through the user interface to match the desired breathing rhythm, confirming successful implementation of the system’s functional design.

## Discussion

EMS-Breathz, the EMS-guided rhythmic breathing system developed in this study, provides a novel technical approach to externally regulate the breathing pattern during radiotherapy. In contrast to conventional respiratory motion-management strategies that primarily monitor or compensate for respiratory motion [3-10], the proposed system is designed to guide breathing patterns by inducing abdominal muscle contractions, thereby providing a structured and repeatable respiratory rhythm based on its operational principle. This approach may be particularly beneficial for patients who struggle to maintain voluntary breath control or consistent respiratory patterns during treatment.

The proposed system offers several technical advantages. First, it provides externally induced physiological modulation, enabling consistent, reproducible breathing cycles by stimulating abdominal muscle contractions. The system is constructed using commercially available fitness-grade electrical stimulation components [11,12], which contribute to a compact and cost-conscious hardware configuration. Its design allows easy integration into existing radiotherapy environments without interfering with standard treatment setups. Furthermore, the system is scalable and expandable. Future versions may incorporate real-time respiratory monitoring or integrate with virtual reality-based visual coaching to further enhance synchronization and patient comfort [6], and may also enable control of radiation delivery timing through the transmission of gating signals to radiotherapy systems [1].

From a theoretical perspective, improved breathing regularity has been reported to reduce respiratory motion variability and improve treatment efficiency under respiratory-gated radiotherapy. Previous studies on stabilized breathing or coaching-based respiratory guidance have demonstrated reductions in respiratory motion variability on the order of approximately 15%-40%, with corresponding improvements in effective beam-on time under gated conditions [16,17]. Although no quantitative physiological measurements were performed in the present technical feasibility study, EMS-guided rhythmic breathing could theoretically contribute to similar benefits by reducing respiratory variability and prolonging stable exhalation phases.

In future applications, several functional enhancements are planned to improve synchronization and clinical usability. First, real-time respiratory monitoring using an optical tracking system [18] could be incorporated to establish closed-loop feedback control, enabling dynamic synchronization between EMS output and the patient’s natural respiratory cycle. Second, the GUI application may support real-time visualization of stimulation output and allow parameter adjustments, such as ON/OFF time, pulse duration, and output current, without interrupting the session. Third, automatic logging of session data and optional visual or auditory cues (e.g., LED blinking or voice prompts such as “breathe in” and “breathe out”) could further enhance reproducibility and patient guidance. In addition, a Bluetooth-based remote-control function for operator use is under consideration.

Although the current prototype was successfully developed and confirmed to function as designed at a technical level, clinical testing has not yet been conducted. Future studies will therefore focus on three areas: 1) evaluating the physiological feasibility of the system in healthy volunteers, particularly in terms of respiratory regularity and comfort, 2) integrating the system with real-time monitoring devices to enable adaptive synchronization, and 3) conducting clinical validation during 4D-CT simulation and gated irradiation to assess potential dosimetric benefits.

Potential limitations of the present design include interpatient variability in EMS responsiveness, contraindications such as pacemaker implantation or skin inflammation, and the need to optimize stimulation parameters such as output current and pulse duration for individual patients. Because the system is based on commercially available electrical stimulation technology, it is generally expected to be safe; however, potential discomfort or sensitivity during use should be systematically evaluated in future studies. Despite these limitations, the proposed system may serve as a foundation for an active, safe, and reproducible respiratory motion management strategy in radiotherapy.

## Conclusions

In conclusion, we developed EMS-Breathz, an EMS-guided rhythmic breathing system to actively regulate the breathing pattern during radiotherapy through rhythmic abdominal stimulation. Its basic operation and functionality were confirmed at a technical level. While clinical validation remains necessary, this system introduces a novel technical direction for improving breathing regularity and treatment reproducibility and establishes a solid foundation for future closed-loop, patient-adaptive respiratory control. Future studies will focus on evaluating feasibility, safety, and clinical applicability in human subjects, with the potential to contribute to more precise, efficient, and patient-friendly respiratory motion management in radiotherapy.
